# Takotsubo Syndrome—Is There a Need for CMR?

**DOI:** 10.1007/s11897-021-00518-x

**Published:** 2021-06-20

**Authors:** Philipp-Johannes Jensch, Thomas Stiermaier, Ingo Eitel

**Affiliations:** 1 University Heart Center Lübeck, Medical Clinic II (Cardiology/Angiology/Intensive Care Medicine), University Hospital Schleswig-Holstein, Ratzeburger Allee 160, 23538 Lübeck, Germany; 2 German Centre for Cardiovascular Research (DZHK), partner site Hamburg/Kiel/Lübeck, Lübeck, Germany

**Keywords:** Takotsubo syndrome, Takotsubo cardiomyopathy, Cardiac magnetic resonance imaging, CMR, MRI, Broken heart syndrome, Apical ballooning syndrome, Stress cardiomyopathy

## Abstract

**Purpose of Review:**

Takotsubo syndrome (TTS) is a transient but severe myocardial dysfunction that has been known for decades and is still to be fully understood regarding its clinical presentations and pathophysiological mechanisms. Cardiac magnetic resonance (CMR) imaging plays a key role in the comprehensive analysis of patients with TTS in acute and follow-up examinations. In this review, we focus on the major advantages and latest evolutions of CMR in diagnosis and prognostication of TTS and discuss future perspectives and needs in the field of research and cardiovascular imaging in TTS.

**Recent Findings:**

Specific CMR criteria for TTS diagnosis at the time of acute presentation are established. In addition to identifying the typical regional wall motion abnormalities, CMR allows for precise quantification of right ventricular and left ventricular (LV) function, the assessment of additional abnormalities/complications (e.g. pericardial and/or pleural effusion, LV thrombi), and most importantly myocardial tissue characterization (myocardial oedema, inflammation, necrosis/fibrosis).

**Summary:**

CMR enables a comprehensive assessment of the entire spectrum of functional and structural changes that occur in patients with TTS and may have also a prognostic impact. CMR can distinguish between TTS and other important differential diagnoses (myocarditis, myocardial infarction) with direct consequences on medical therapy.

## Introduction

Takotsubo syndrome (TTS), which typically mimics acute coronary syndrome (ACS) with acute occurring symptoms such as angina pectoris or dyspnoea [1, 2], new findings in the electrocardiogram (ECG) (e.g. pathologic ST-segment, T-waves or QTc-segments) [3–5] and biochemical rise in cardiac markers (troponin, brain natriuretic peptide) [6, 7], was firstly described at the beginning of the 1990s in Japan and likewise later in Europe and worldwide [8–10]. As its unique attribute, TTS has a distinctive pattern of reversible left ventricular (LV) regional myocardial wall movement abnormalities (RWMA), which are not likely to be assigned to a specific coronary artery territory and mostly lack obstructive coronary artery disease (CAD) [11]. The reversibility of the severe myocardial dysfunction usually occurs within weeks to months but is accompanied by potential life-threatening complications in the acute phase like severe heart failure and cardiogenic shock [12, 13] as well as arrhythmias [14–16]. Various diagnostic criteria for TTS have been published and have recently been incorporated by international Takotsubo experts (InterTAK) (Table 1):

**Table 1:** InterTAK diagnostic criteria for TTS (modified from Ghadri et al. [10])

	Diagnostic criteria
1	Transient regional wall movement abnormalities of left and/or right ventricle (hypo-, dys- or akinesia), in the majority beyond a single epicardial vascular distribution Rare cases with RWMA in the subtended myocardial territory of a single coronary artery (focal TTS) exist
2	A detectable emotional and/or physical trigger is elicitable, but not obligatory
3	Neurologic disorders (stroke, transient ischaemic attack, haemorrhage) or pheochromocytoma may serve as triggers
4	New ECG abnormalities (ST-segment elevation, ST-segment depression, T-wave inversion, QTc prolongation), thus rare cases without ECG changes
5	Elevation of cardiac biomarkers (primarily troponin, creatine kinase, brain natriuretic peptide (BNP))
6	Significant coronary artery disease (CAD) is not a contradiction
7	No evidence of infectious myocarditis (exclusion via CMR is obligatory)
8	Predominant affection of post-menopausal woman

**Fig. 1:**
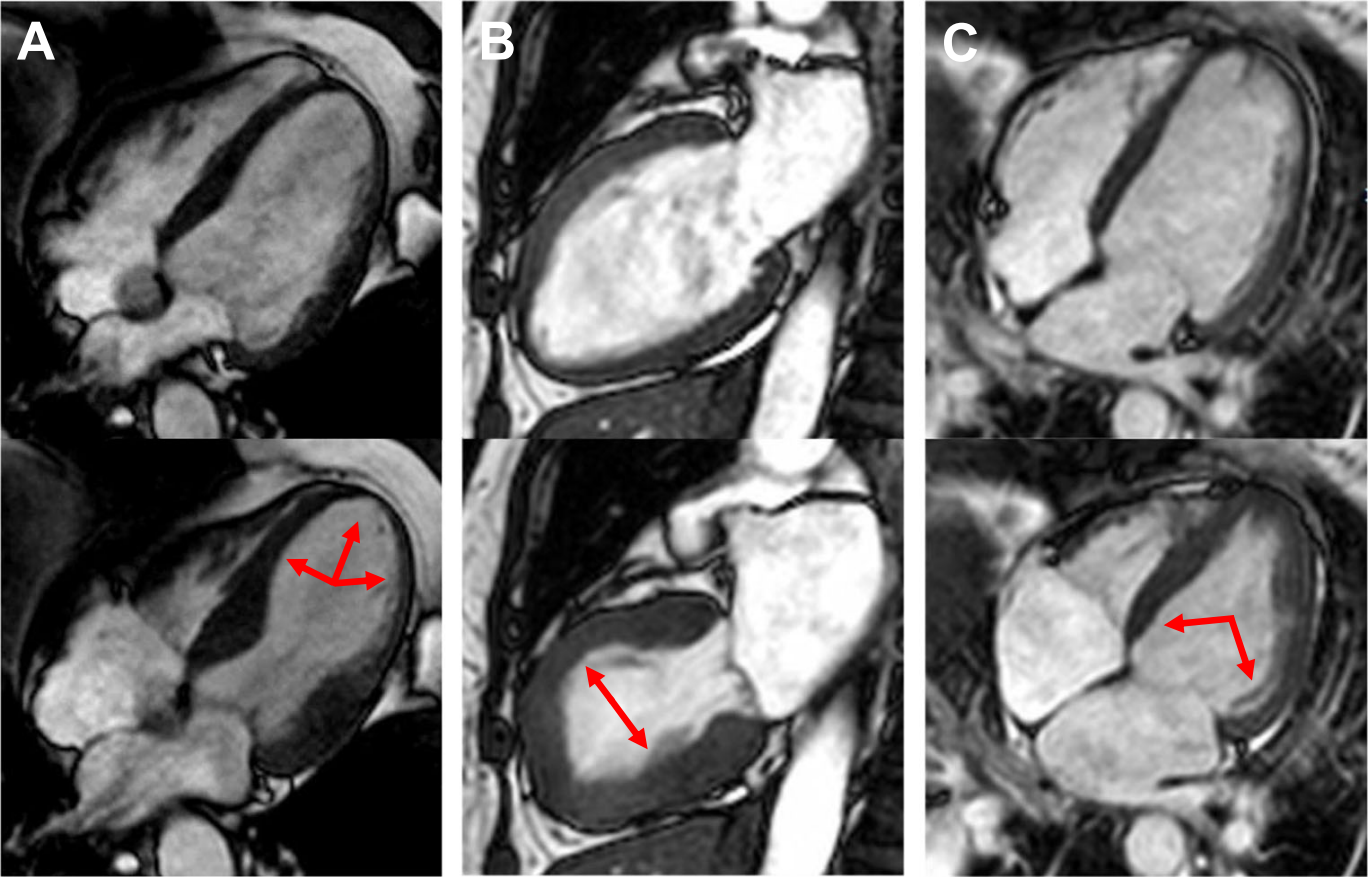
CMR imaging with apical (**A**), midventricular (**B**) and basal (C) Takotsubo syndrome. Top row = end-diastole; bottom-row = end-systole

Referring to the latest guidelines of the European Society of Cardiology (ESC), TTS is no longer classified as myocardial infarction with non-obstructive coronary arteries (MINOCA) [17] but rather regarded as its own entity that is diagnosed by ventriculography, echocardiography and CMR [18]. During the rule out of CAD in the acute clinical presentation, TTS is often diagnosed in the LV-angiography with pathognomonic RWMA. In the first case report, TTS showed the typical systolic apical ballooning type with akinetic apical and midventricular but hypercontractile basal segments, which reminded the Japanese researcher of the traditional octopus trap “Takotsubo” [8]. Of note, several ballooning patterns have been described in the following course of research and better knowledge of the syndrome [19]. Depending on the involved areas with patterns of myocardial stunning and hypercontractility, one can differentiate three main morphological types, visualized in Fig. 1, which are completed by relatively rare cases of focal ballooning.

Furthermore, large registers validate the predominance of 80% of patients being postmenopausal women [20, 21]. Until today, no gratifying theory could prove the pathophysiology of TTS, but in most cases, an emotional trigger is evident and experts consider sympathetic stimulation with catecholamine excess leading to impaired coronary microcirculation [22] plus activated specific cerebral regions as the likeliest explanation [23]. The InterTAK classification of Ghadri et al. further diversifies the type of triggering event, with two-thirds of all patients experiencing acute emotional (class I) and less often physical (class II) stress but also with one-third of patients presenting without trigger events (class III) [24] (Table 2).

**Table 2:** InterTAK classification of TTS based on triggering events, modified from Ghadri et al. [24]

Class I	TTS related to emotional stress
Class II	TTS related to physical stress
Class II a	Secondary to physical activities, medical conditions or procedures
Class II b	Secondary to neurological disorders
Class III	TTS without identifiable trigger

In the first period, major adverse cardiac events (MACE) such as acute heart failure, arrhythmias and cardiogenic shock are in frequency and complexity comparable to ST-elevation myocardial infarction (STEMI) with mortality rates of 3.5–5% in hospital [12, 25, 26] and 10–12% per year [1, 27]. In the clinical course, however, the characteristic myocardial functional limitations such as apical, midventricular or basal ballooning, the electrocardiographic abnormalities and the myocardial damage with increased biomarkers disappear completely within weeks in most of the cases [23, 27, 28].

## Coronary Angiography and Ventriculography

Due to its clinical presentation as STEMI or high-risk non-STEMI, TTS patients mandatorily need coronary angiography for the exclusion of myocardial infarction according to current guidelines [23, 29]. However, interventionalists have to identify a myocardial perfusion-contraction mismatch in order to differentiate AMI and TTS reliably, since 23% of TTS patients have obstructive CAD and even 41% have unobstructed CAD [30, 31]. Mandatorily, a culprit atherosclerotic plaque rupture, thrombus formation or coronary dissection should not be sufficient for the explanation of RWMA. Subsequently additional ventriculography is the obvious pathway for the visualisation of typical RWMA if TTS is present. According to Desmet et al., one can distinguish TTS from anterior STEMI in patients with apical ballooning if a small apical area with preserved contractility is detectable (the “apical nipple sign”) [32].

## Echocardiography

Transthoracic echocardiography (TTE) is the image modality of choice in the acute presentation of ACS or TTS to assess amendments of the cardiac function and morphology. For instance, RWMA and RV-involvement (biventricular ballooning, “reverse McConnell sign”) in the acute setting as well as mechanical complications (left ventricular outflow tract obstruction (LVOTO), mitral regurgitation (MR), wall rupture), LV thrombi, pericardial effusion and pulmonary artery pressure in the subacute phase and later course of the disease [33–35]. Furthermore, TTE enables to objectify the circumferential impairment of LV longitudinal and radial strain via speckle tracking, which seems to persist longer than RWMA and reduced LVEF [34, 36–38].

Subsuming, echocardiographic findings of high-risk TTS patients with adverse in-1hospital outcomes are the following: LVEF < 35% and low cardiac output, RV involvement, LVOTO, MR > 2 +, LV thrombi, pericardial effusion and wall rupture [37, 39].

Notably, the diagnosis of rare TTS-patters beyond apical or midventricular forms is highly challenging and not reliably possible without other imaging modalities. During follow-up, TTE is broadly used to monitor the recovery of LV-function, the regression of RWMA and to detect the occurrence of mechanical complications [40].

## Cardiac Magnetic Resonance

Whilst echocardiography being the most accessible and low cost in use and coronary plus ventricular angiography being performed to rule out acute myocardial infarction (AMI) in the acute phase, CMR displays the reference standard for the concise assessment of left ventricular (LV) and right ventricular (RV) volume and functions as well as detection of wall movement abnormalities (RWMA) [20, 41]. Furthermore, other pathologies like pericardial or pleural effusion and intracardial thrombi can be detected. CMR can be utilised in the post-acute phase after clinical stabilisation and is used for the follow-up analysis after several months.

Recently, specific CMR criteria for TTS diagnosis at the time of acute presentation were established which include the combination of typical RWMAs, oedema and the absence of evidence of irreversible tissue injury (late gadolinium enhancement (LGE)) [20].

Moreover, CMR is a potent alternative if echocardiography generates suboptimal image quality, if urgent information about the ventricular morphology, potential differential diagnosis or complications of acute ischaemic cardiac symptoms are needed.

CMR is uniquely suited for the evaluation of patients with TTC as gold standard for functional imaging and its unique ability of myocardial tissue characterisation. Regarding the CMR protocol in patients with suspected TTS (Table 3), we outlined functional imaging in grey and myocardial tissue characterisation in blue.

**Table 3:** CMR protocol in patients with suspected TTS, modified according to Ojha et al. [42]

Protocol	Sequence	Views	Application	
1. Scout	Non-gated bSSFP	Axial, coronal, sagittal	Extracardiac findings (pericardial and/or pleural effusion)	Mandatory
2. Morphology and function	bSSFP (ECG and respiratory gated) or FSE	Short axis and long axis (two-chamber view, three-chamber view with LVOT and four-chamber view)	Characteristic contraction patterns with RWMA (hyper-,dys- or akinesis; apical, midventricular, basal or focal ballooning) and involvement of RV	Mandatory
3.Inflammation and oedema	T2bb FSE	Short axis of LV	Distinguishment of myocarditis and AMI Oedema: SI > 1.9 times of skeletal muscle	Mandatory
4. Native T1 mapping	MOLLI or shMOLLI	Short-axis slices of basal, mid- and apical LV	Quantification of oedema, inflammation and myocardial injury	Optional
5. T2 mapping	Single-shot bSSFP	Short-axis slices of basal, mid and apical LV	Quantification of oedema and inflammation	Mandatory
6. Strain / Feature tracking	bSSFP	Short-axis end-diastolic and end-systolic	Quantification of regional and global strain patterns (transient myocardial dyssynchrony, preserved systolic torsion)	Optional
7. Perfusion, first pass of intravenous Gd	Saturation recovery sequence	Short axis slices of basal, mid and apical LV	Subendocardial microvascular dysfunction	Optional
8. EGE (< 3 min after infection)	PSIR at TI 550ms	Multiple short-axis slices or long axis 2-, 3- and 4-chamber views covering entire LV	Inflammation (hyperaemia, capillary leak, diffuse oedema)	Optional
9. LGE (5–10 min after infection)	PSIR at TI 200 ms	Same as in previous EGE	Irreversible cell injury (fibrosis, necrosis) Absence in TTS (using +5 SD thresholds)	Mandatory
10. Post-contrast T1 mapping (at least 15 min after Gd)	MOLLI	Same slices as native T1	Calculation of extracellular volume	Optional

*bSSFP* balanced steady-state free precession; *FSE* fast spin echo; *T2bb* T2-weighted block blood imaging; *SI* signal intensity; *RV* right ventricle; *(sh)MOLLI* (shortened) modified Look locker inversion recovery; *PSIR* phase-sensitive (black blood T2w) short tau inversion recovery; *Gd* gadolinium; *EGE* early gadolinium enhancement; *LGE* late gadolinium enhancement; *TI* time inversion; *ms* milliseconds

## Functional Imaging

In accordance with the recent published standardized protocols by the Society for Cardiovascular Magnetic Resonance (SCMR), the initial scout should be followed by balanced steady-state free precession (bSSFP) or fast spin echo (FSE) sequences for image acquisition in short axis and long axis (two-chamber view, three-chamber view with LVOT and four-chamber view) orientations [43]. These planes should enable to acquire a high-resolution illustration of the left ventricular (LV) and the right ventricular (RV) chamber with its potential anomalies.

Concerning TTS, one can visualize the typical RWMA of the LV with a major decrease of the ejection-fraction (EF) due to hypokinesia with ballooning of the apical region (82%), the midventricular region (17%), the basal region (1%) or of focal regions in very rare cases (Fig. 1) [20]. Referring to the current data, the EF and WMA show a normalization to the baseline value prior the TTS within months [20, 40].

At the acute phase, it is recommended to quantify the LV volume, the LV mass, the cavity diameter and the LV wall thickness [44]. Several possible theories on the pathophysiological concepts of TTS RWMA and transient reduction of EF exist; however, they are yet to be proven and not being further discussed in this review.

Another crucial aspect is the detection of RV involvement (RVi) in TTS patients, which commonly has been associated with prolonged hospitalization and higher rate of MACE [20, 45–47]. In the study of Citro et al., 13% of the TTS population had RVi in echocardiography with a significantly higher mortality, but Scally et al. investigated that CMR could double the detection rate of RV-RWMA [33, 45].

Stiermaier et al. firstly described a transient reduction of the left atrial (LA) emptying fraction (EF) in the acute phase of TTS, with an LA-EF improvement from 42 to 51% after 3 to 5 months [48]. Hence, the reduction auf die LA-EF is closely connected to the transient impairment of the LVEF and its diastolic parameters. Until now, reduction of LA-EF in TTS tends to be an additional feature together with an impairment LV- and RV-EF.

**Fig. 2:**
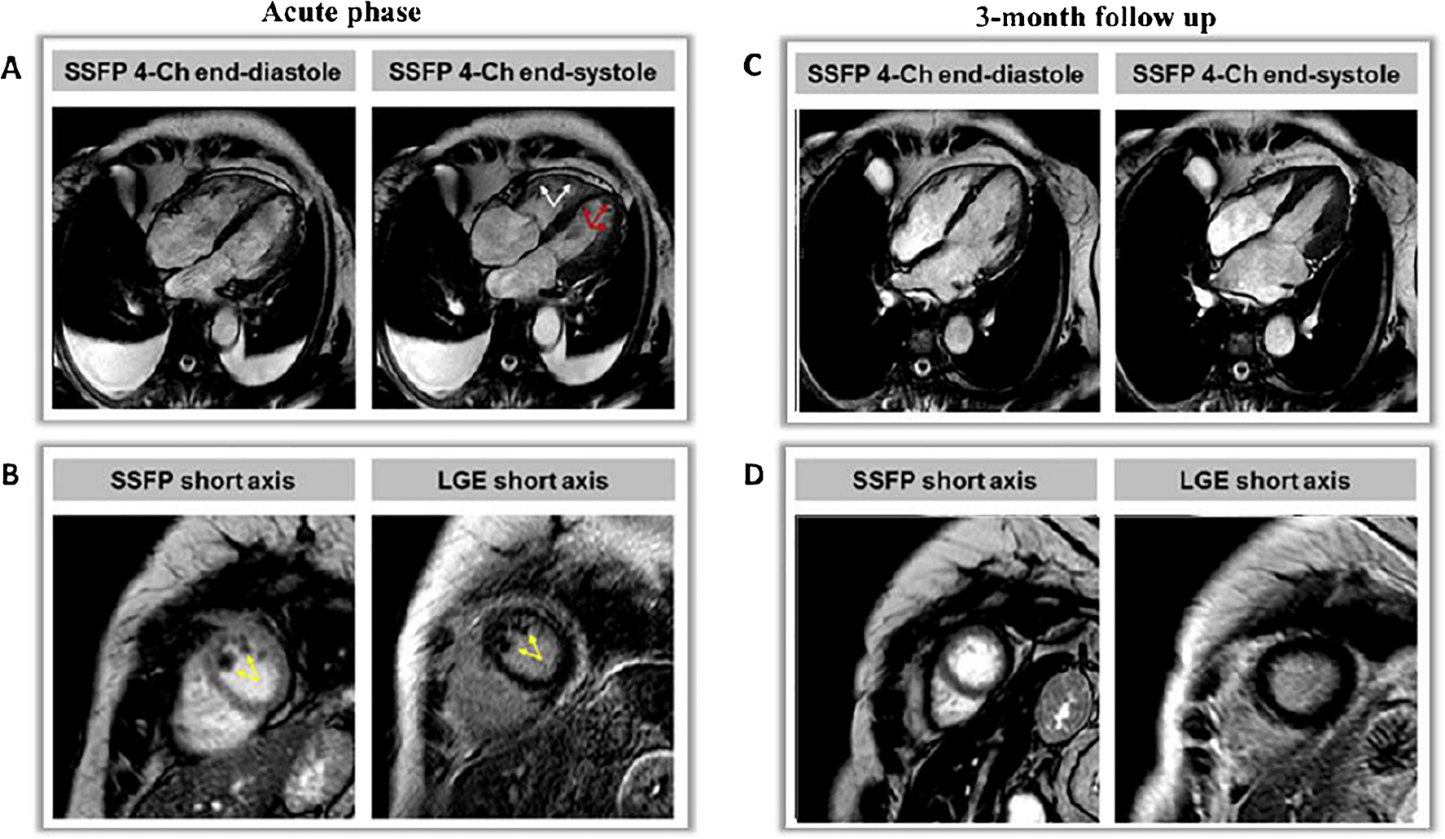
CMR imaging of a patient with TTS complications. Acute phase: Four-chamber views of biventricular ballooning with reduced LVEF (< 30%) and bilateral pleural effusion (**A**) and short axis views of LV thrombi (**B**); 3-month follow-up: recovery of LVEF (**C**) and disappearance of LV thrombi (**D**)

Moreover, major complications like LV thrombi, pericardial or pleural effusion and very rare septum or free wall ruptures can easily be detected by the use of CMR [41]. Despite their relatively low incidence according to Santoro et al. (2.2%), LV thrombi obviously have a huge prognostic impact in TTS, mainly if the treatment with oral anticoagulation [49] is delayed or missed and strokes occur (Fig. 2).

Latterly, the method of feature-tracking (FT) in CMR has become a promising parameter for the measurement of regional deformation like dyssynchrony and rotational parameters in all four heart chambers, corresponding to speckle tracking echocardiography [39, 50–52]. Simplified, bSSFP short-axis end-diastolic (ED) and end-systolic (ES) images are used to trace endocardial and epicardial borders and its movement semiautomatic in at least three slices. If RWMA appear, the physiological, equal myocardial strain is postulated to change in radial and circumferential manners.

**Fig. 3:**
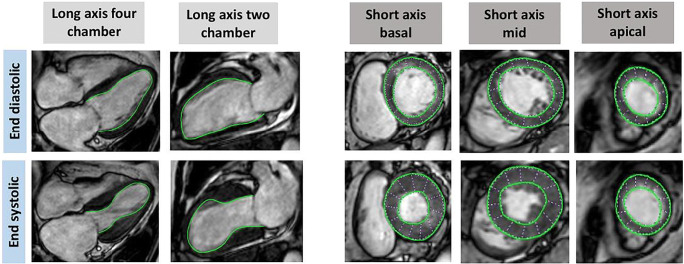
CMR FT end-diastolic and end-systolic in long-axis four and two chambers as well as short-axis basal, midventricular and apical segments

Notedly, the usage of CMR-FT enabled the identification of several new parameters of prognostic relevance, such as transient myocardial dyssynchrony in the acute setting and preserved systolic torsion in the follow-up. Referring to Backhaus et al., especially patients with the typical apical ballooning, biventricular ballooning, reduced LVEF or malignancies were vulnerable for dyssynchrony, with a non-significant but trend on higher mortality. Additionally, radial- and circumferential strain allowed a reliable discrimination between apical and midventricular ballooning [53] (Fig. 3).

However, only few studies investigated the prognostic value of LV- and RV-FT. Accordingly, TTS patients show comparable strain-pattern like STEMI patients but with significantly lower values. Regarding the LV, radial and circumferential strain showed no predictive value, whereas the reduction of global longitudinal strain was identified as a potential parameter for long-term risk stratification [52]. Recently, Stiermaier et al. showed that longitudinal strain assessed by FT is associated with adverse clinical outcome in TTS but is outperformed by comorbidities such as diabetes mellitus or atrial fibrillation in multivariable analysis [54]. Although the impairment of the RV-strain in CMR-FT is yet to show additional predictive value, the latest results of Stiermaier et al. suggest at least a superiority of stratification via a RV strain threshold (of − 17,24%) compared to visual RV evaluation only [39].

Latest investigations of both atria via CMR-FT showed at least a transient impairment of the LA reservoir and conduit function in acute TTS with an independent prediction of mortality.

Meanwhile, the method of CMR-FT of the LV shows a high intra- and inter-observer reproducibility [55]. Compared to speckle-tracking as a similar technique in echocardiography, CMR-FT is more accurate and reliable [40]. Nonetheless, the clinical utility and prognostic impact of CMR-FT is yet to be validated in prospective, multicentric studies for the routine application in a clinical setting.

Subsuming the outlined potencies of CMR concerning functional changes in TTS-patients, we conclude the following: by performing bSSFP or FSE for cine imaging, [1] typical hypokinesia and RWMA can solidly be determined; [2] additional prognostic value of RVi and LA-EF can be generated and can reproducibly be measured; [3] life-threatening complications become obvious (e.g. LV thrombus) and can be easily detected during the acute CMR Scan; and [4] strain-analysis via FT is promising and may open new prognostic perspectives in TTS.

## Myocardial Tissue Characterization

The enabling of a multiparametric myocardial tissue characterisation is one of the most advantageous capabilities of CMR to discriminate reversible from irreversible myocardial injury. Therefore, by the usage and absence of late gadolinium enhancement (LGE), one can preclude myocarditis or an ischaemic myocardial infarction (e.g. with spontaneous lysis of thrombus), which is, according to the latest literature, one of the main diagnostic criteria of TTS [56, 57]. Concurring to the recommended SCMR protocol, bSSFP and phase-sensitive inversion-recovery (PSIR) are performed for fibrosis/scar imaging. With a delay of 5 to10 min after gadolinium injection, the initial cine imaging is repeated with short inversion time (TI) of about 200 ms [43].

In myocarditis, LGE pictures as patchy myocardial necrosis or/and fibrosis. However, focal LGE was detected in TTS-patients once low signal intensity (SI) thresholds of three standard deviations (SD) from a healthy myocardial reference contour were used. These findings are not reproducible when high SI thresholds are applied and fade out within weeks, which reflect their transient character [20, 58]. Some studies suggest that the occasional appearance of LGE in TTS might be related to increased interstitial water with delayed washout of the gadolinium [59]. An immunohistologic analysis of Rolf et al. found increased levels of extracellular collagen-1 as a sign of integrity loss of myocytes and transient fibrosis [60].

**Fig. 4:**
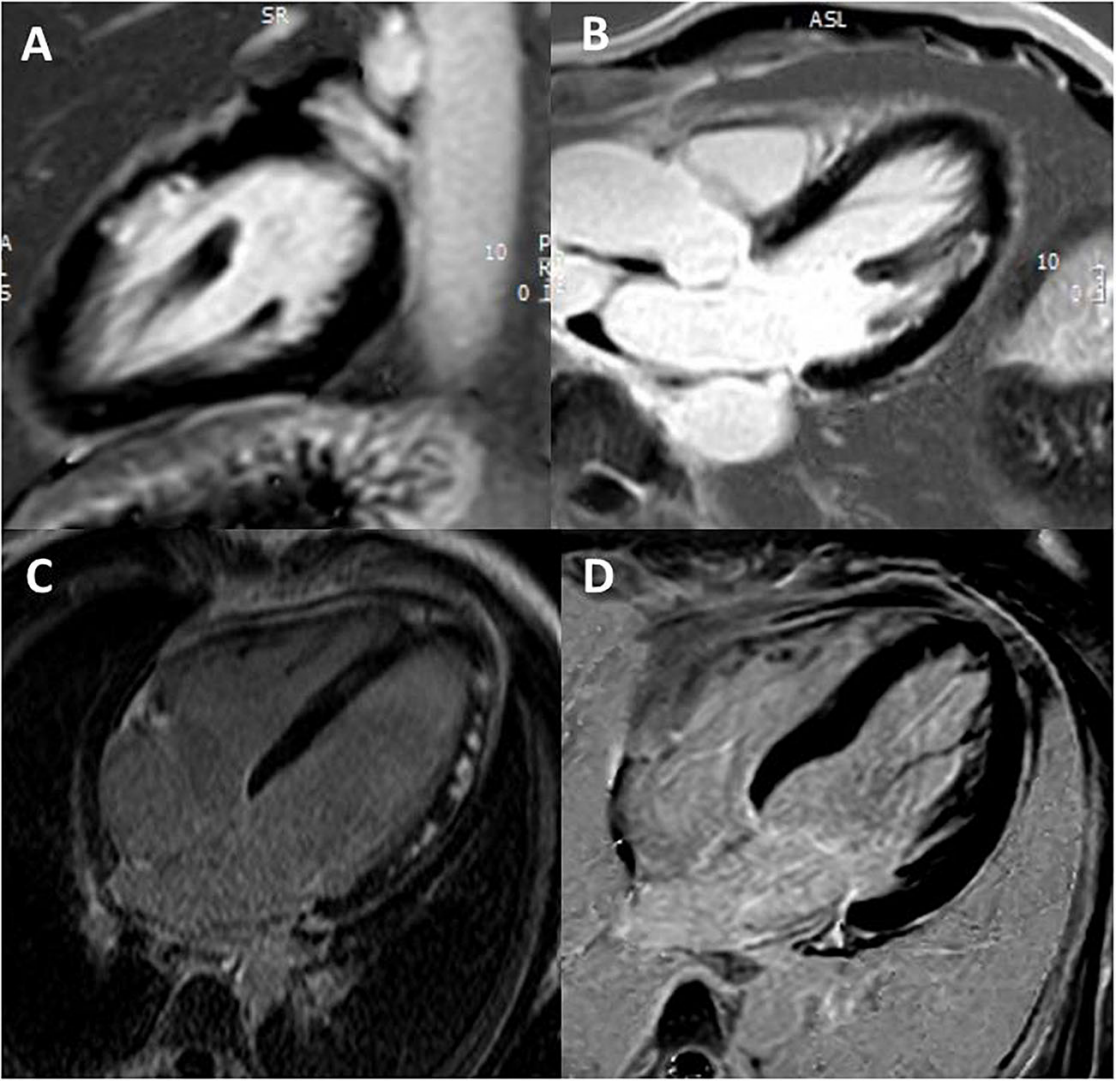
Different LGE patterns in suspected MINOCA. **A** and **B** Myocardial infarction with spontaneous lysis of thrombus with subendocardial ischemic LGE of the lateral wall in 2-chamber (**A**) and 3-chamber (**B**) views**. C** Patchy, subepicardial non-ischemic LGE in a patient with myocarditis (4-chamber view). **D** Absence of irreversible tissue injury (LGE) when SI threshold of 5 SD is used in a patient with TTS (4-chamber view)

Recapitulated, the absence of LGE at high SI thresholds remains a main diagnostic criterion for TTS, noteworthy that patchy LGE at lower SI might occur in rare cases. Nonetheless, until today no significant differences concerning clinical outcome of TTS-patients without- or with LGE of three SD have been validated in prospective registries [20, 59] (Fig. 4).

One of the main features of TTS is acute inflammation of unknown mechanisms with an increment of intra- and extracellular fluid causing oedema and cellular injury with troponinaemia. A possible explanation is transient ischaemia or increased myocardial wall movement stress which rarely leads to persisting cellular damage compared to STEMI or myocarditis. Characteristically, in TTS patients, oedema is not limited to one coronary coverage area; it is mostly diffuse and vanishes alongside the recovery of myocardial contractility within weeks. Thus, oedema is a key diagnostic feature for the determination of severity and extent of myocardial stunning [61].

**Fig. 5:**
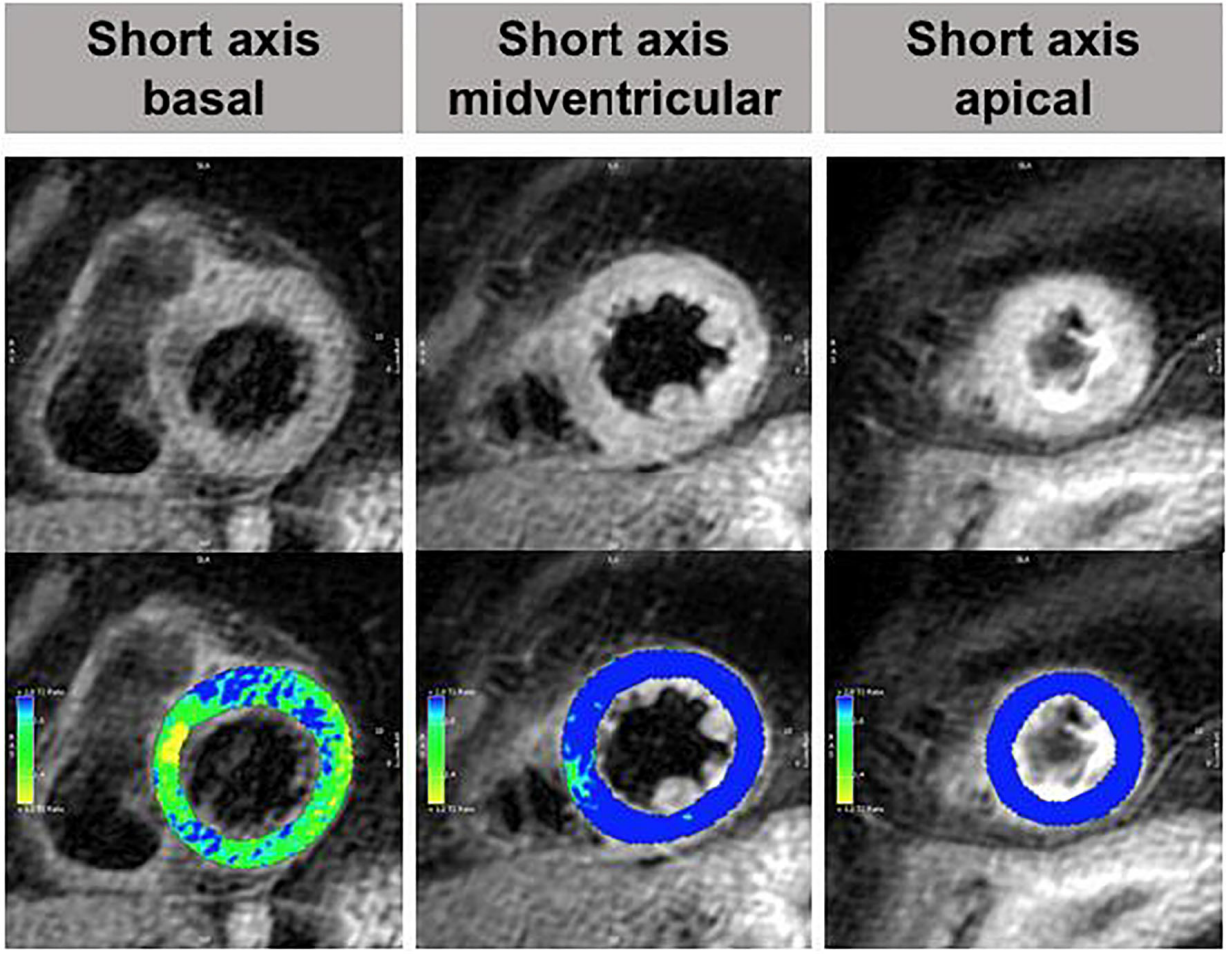
CMR imaging in a patient with typical apical TTS. Top row: T2-weighted short tau inversion recovery images demonstrating normal signal intensity of the basal myocardium but global oedema of the mid- and apical segments with impaired systolic function. Bottom row: Computer-aided signal intensity analysis of the oedema images (blue indicates a signal intensity ratio of myocardium to skeletal muscle ≥ 1.9 equivalent to oedema; green/yellow indicates a normal signal intensity ratio < 1.9)

Several CMR techniques for the visualisation of oedema, such as “T2-weighted block blood imaging” (T2bb) with equal suppression of blood and fat, native T1 mapping or T2 mapping, exist. In T2bb a SI ratio (between myocardium and skeletal muscle) equal to or greater than 1.9 is considered as oedema. Meanwhile, techniques for a more precise and objective assessment such as motion correction algorithms of parametric T1 and T2 mapping with bail out of saturation- and motion artefacts compared to routine T2 sequences gain wide consent [62] (Fig. 5).

Noteworthy, the field of mapping techniques for the detection of damaged myocardial areas without additional gadolinium contrast is rapidly developing with a huge variety of pulse sequences. For image acquisition, one should consider the timing of sequences prior to gadolinium application and the measurement of haematocrit within 24 h for the accurate measurement of extracellular volume fraction (ECV).

**Fig. 6:**
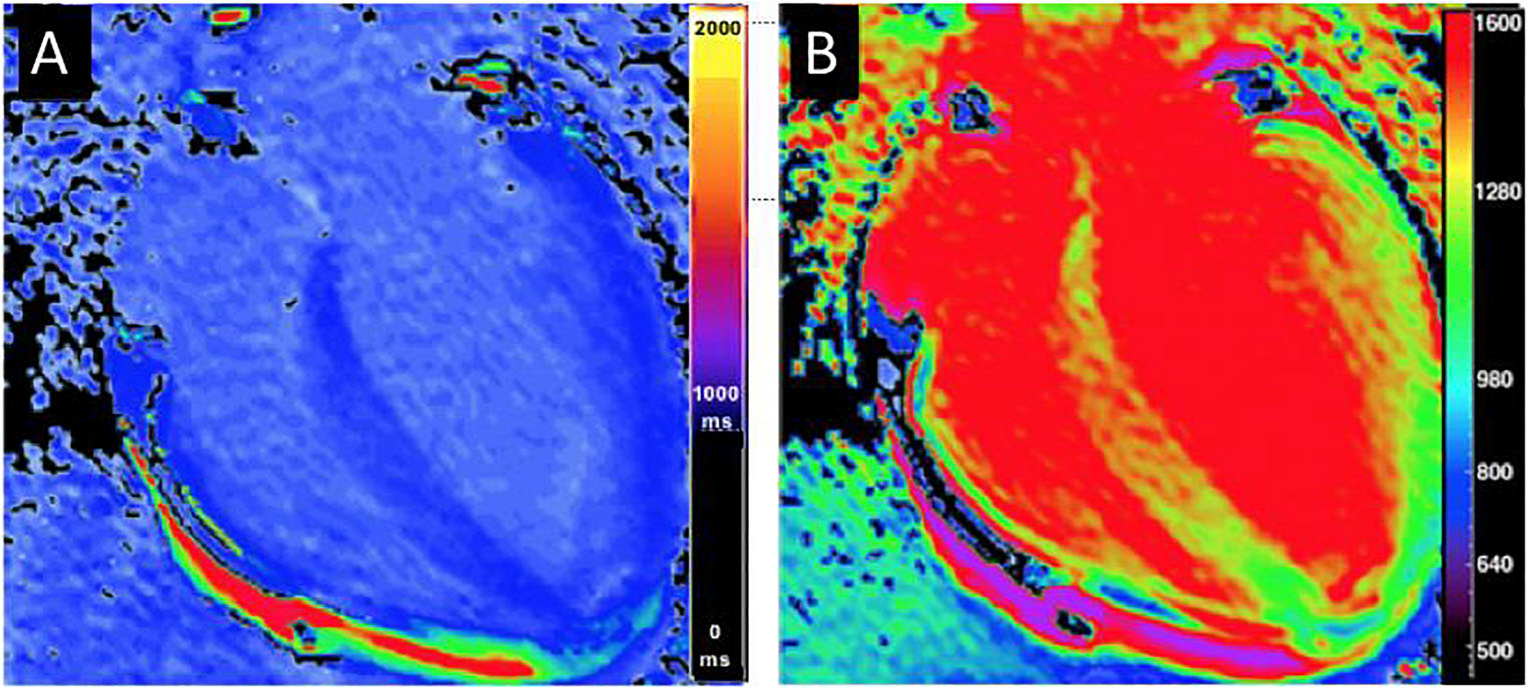
T1 mapping in Takotsubo syndrome. Colour-coded native (**A**) and post-contrast (**B**) T1 maps using ShMOLLI with regional myocardial involvement

For T1 mapping, modified Look locker inversion recovery (MOLLI) or shortened MOLLI (shMOLLI) are recommended (Fig. 6).

T2 mapping is performed via single-shot bSSFP with different T2 prep times, gradient and spin echo (GraSE) and T2-weighted (T2w) via “black blood T2w short tau inversion recovery” (STIR) [43].

In the acute phase, some small studies suggest significantly higher T1 and ECV values in areas of RWMA in TTS patients which persisted in the follow-up even after normalisation of LVEF. Likewise, T2 values were initially risen but normalised completely [62]. Other studies showed different results with persisting T1, T2 and ECV values in the follow-up [63, 64]. At the present time, mapping techniques need further research in large clinical trials for a valid statement on standardisation of sequences and cut-off values in guidelines and a consecutively ubiquitous implementation in clinical practise.

Aggregating the stated capabilities of CMR in tissue characterisation of TTS patients, we suggest [1] the constant usage of LGE with a high SI threshold of five SD for the exclusion of relevant myocardial necrosis, [2] the routine visualisation of oedema in the acute setting and [3] the liberal acquisition of mapping sequences for scientific reasons.

## Conclusion

CMR should be used whenever possible in the acute setting and follow-up for diagnosing and prognostication of TTS patients. The main potencies are the acquisition of valid and detailed information about contractility abnormalities, structural complications and tissue characterisation with absence of LGE and visualisation of oedema as a pathognomonic key feature in TTS. In detail, typical RWMA with ballooning patterns and myocardial oedema as a hint of reversible tissue injury are commonly visualised while LGE as proof of irreversible tissue injury is absent. However, the pathophysiological mechanisms underlying myocardial oedema remain unclear and subtle fibrosis may rarely be seen through LGE in TTS. Additionally, CMR is the validated gold standard for the detection of RV-TS and the detection of biventricular ballooning, which has incremental impact on morbidity and clinical outcome.

Additionally, major information for the evaluation of potential differential diagnosis of acute ischaemic cardiac symptoms ((peri-)myocarditis, myocardial infarction) or possible complications (thrombi, septum- or free wall ruptures) can easily be generated and converted and are important for therapeutic decision making.

## Future development

Notwithstanding the huge number of potential pathophysiological mechanisms underlying TTS, a gratifying explanation is yet to be proven. Further research in all fields including cardiovascular imaging is needed to better understand the pathophysiology and clinical spectrum of TTS. Such better knowledge regarding underlying myocardial and microvascular mechanisms is important before clinical trials with evidence-based therapeutic algorithms for treatment of TTS patients can be performed.

Regarding clinical trials with CMR-imaging, promising opportunities for research exist in the wide field of FT and further validation and standardisation of mapping techniques. The occurrence of diffuse fibrosis as detected by T1 mapping techniques and its potential prognostic impact may be future research targets. Moreover, it needs to be assessed if there are specific mapping markers of TTS like specific T1 times and pattern in comparison to other relevant differential diagnosis like myocardial infarction or myocarditis.

Most recently the pandemic of “severe acute respiratory syndrome coronavirus type 2” (SARS-CoV-2) as coronavirus disease 2019 (COVID-19) has led to a global health crisis. Myocardial injury emerged as a major predictor for worse outcome [65, 66] and clinical symptoms of COVID-19 (e.g. hypoxaemia, septic status, metabolic acidosis and emotional stress) are likely to trigger a secondary TTS in 4.14% of patients as reported by Hegde et al. [67, 68]. Regarding 120 million infected patients and 2.65 million deaths worldwide since December 2019 (Dashboard Johns Hopkins University March 11, 2021), there is a possible number of up to 4.97 Million patients which widely undiscovered faced the acute complications of developing TTS. Conclusive CMR data of COVID-19-induced TTS in the acute- and post-acute phase is unavailable so far and a huge and interesting field for further investigations. Up to date, there are only occasional CMR studies focussing on general COVID-19-related myocardial changes [69] and more data about COVID-19-induced TTS is urgently needed.
